# Aqua­[*N*′-(3-eth­oxy-2-oxidobenzyl-κ*O*)furan-1-carbohydrazidato-κ^2^ *N*′,*O*]dioxido­molybdenum(VI)–4,4′-bipyridine (2/1)

**DOI:** 10.1107/S1600536811017260

**Published:** 2011-05-14

**Authors:** Ngui Khiong Ngan, Richard Chee Seng Wong, Kong Mun Lo, Seik Weng Ng

**Affiliations:** a Department of Chemistry, University of Malaya, 50603 Kuala Lumpur, Malaysia

## Abstract

The Mo^VI^ atom in the title co-crystal, [Mo(C_14_H_12_N_2_O_4_)O_2_(H_2_O)]·0.5C_10_H_8_N_2_, is *O*,*N*,*O*′-chelated by the deprotonated Schiff base and coordinated by the oxide and water O atoms in an octa­hedral geometry. The five-membered chelate ring is planar (r.m.s. deviation = 0.019 Å), but the six-membered chelate ring is puckered (r.m.s. deviation = 0.108 Å). Two mononuclear mol­ecules are linked across a center of inversion by an O—H_water_⋯O hydrogen bond; adjacent dinuclear units are linked by an water–4,4′-bipyridine O—H⋯N hydrogen bond, generating a linear chain structure. The 4,4′-bipyridine mol­ecule is disordered over two positions in a 1:1 ratio.

## Related literature

For a related Mo^VI^O_2_–4′,4-bipyridine adduct, see: Dinda *et al.* (2006 ▶).
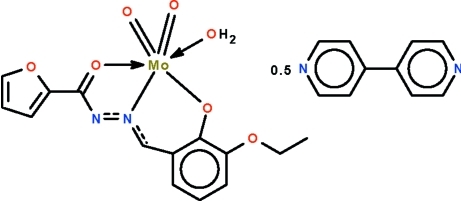


## Experimental

### 

#### Crystal data

[Mo(C_14_H_12_N_2_O_4_)O_2_(H_2_O)]·0.5C_10_H_8_N_2_
*M* *_r_* = 496.30
Triclinic, 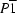

*a* = 7.9237 (1) Å
*b* = 10.1869 (1) Å
*c* = 13.3215 (2) Å
α = 78.7841 (5)°
β = 78.4605 (5)°
γ = 69.5728 (5)°
*V* = 978.15 (2) Å^3^
*Z* = 2
Mo *K*α radiation
μ = 0.72 mm^−1^
*T* = 100 K
0.2 × 0.2 × 0.2 mm

#### Data collection

Bruker SMART APEX diffractometer
Absorption correction: multi-scan (*SADABS*; Sheldrick, 1996 ▶) *T*_min_ = 0.649, *T*_max_ = 0.746
9175 measured reflections
4445 independent reflections
4266 reflections with *I* > 2σ(*I*)
*R*_int_ = 0.019

#### Refinement

*R*[*F* ^2^ > 2σ(*F* ^2^)] = 0.026
*wR*(*F* ^2^) = 0.076
*S* = 0.98
4445 reflections
275 parameters
24 restraints
H atoms treated by a mixture of independent and constrained refinement
Δρ_max_ = 0.73 e Å^−3^
Δρ_min_ = −0.72 e Å^−3^

### 

Data collection: *APEX2* (Bruker, 2009 ▶); cell refinement: *SAINT* (Bruker, 2009 ▶); data reduction: *SAINT*; program(s) used to solve structure: *SHELXS97* (Sheldrick, 2008 ▶); program(s) used to refine structure: *SHELXL97* (Sheldrick, 2008 ▶); molecular graphics: *X-SEED* (Barbour, 2001 ▶); software used to prepare material for publication: *publCIF* (Westrip, 2010 ▶).

## Supplementary Material

Crystal structure: contains datablocks global, I. DOI: 10.1107/S1600536811017260/jh2290sup1.cif

Structure factors: contains datablocks I. DOI: 10.1107/S1600536811017260/jh2290Isup2.hkl

Additional supplementary materials:  crystallographic information; 3D view; checkCIF report

## Figures and Tables

**Table 1:** Hydrogen-bond geometry (Å, °)

*D*—H⋯*A*	*D*—H	H⋯*A*	*D*⋯*A*	*D*—H⋯*A*
O1w—H11⋯N3	0.83 (1)	1.86 (1)	2.689 (3)	174 (3)
O1w—H12⋯N1^i^	0.84 (1)	1.97 (1)	2.794 (2)	167 (3)

Symmetry code: (i) 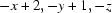.

**Fig. 1.**
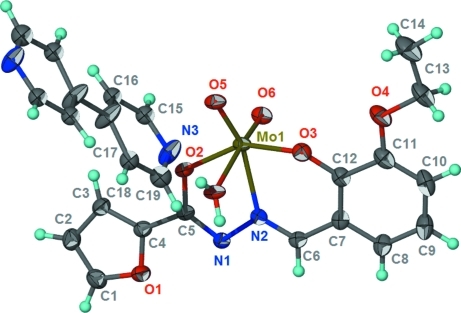
Thermal ellipsoid plot (Barbour, 2001) of MoO2(H2O)(C14H12N2O4).0.5C10H10N2 at the 70% probability level; hydrogen atoms are drawn as spheres of arbitrary radius. The disorder in the 4,4'-bipyridine molecule is not shown.

## supplementary crystallographic information

### Comment

The Schiff bases that are synthesized by condensing salicylaldehyde (and its substituted analogs) with aroylhydrazides (and their substituted analogs) function as terdentate *O*,*N*,*O*'-chelates to a wide range of metal ions. A large number of metal derivatives have been reported; in octahedral systems, the ligand generally exists as a doubly-deprotonated species that chelates in a *fac* manner. A dioxomolybdenum(VI) derivative is known in which 4,4'-bipyridine binds to two metal atoms (Dinda *et al.*, 2006). In the present study, a furan-type of Schiff base leads to a water-coordinated derivative in which 4,4'-bipyridine interacts indirectly, through the water molecule, in an outer-sphere coordination mode. The Mo^VI^ atom in the co-crystal, MoO_2_(H_2_O)(C_14_H_12_N_2_O_4_)^.^0.5C_10_H_10_N_2_, is *O*,*N*,*O*'-chelated by the deprotonated Schiff base and coordinated by the oxo and water O atoms in an octahedral geometry (Scheme I, Fig. 1). The five-membed chelate ring is planar [r.m.s. deviation 0.019 Å] but the six-membered chelate ring is puckered [r.m.s. deviation 0.108 Å]. Two mononuclear molecules are linked across a center-of-inversion by an O–H_water_···O hydrogen bond; adjacent dinuclear units are linked by an *O*–H_water_···N_4,4'-bipyridine_ hydrogen bond to generate a linear chain structure (Table 1). The 4,4'-bipyridine molecule is disordered over two positions in a 1:1 ratio.

### Experimental

3-Ethoxysalicylaldehyde (0.166 g, 1 mmol) and 2-furoylhydrazide (0.120 g, 1 mmol) were condensed in methanol (100 ml). The solution was heated to give a yellow coloration. The cool solution yielded the desired Schiff base as a yellow compound. The ligand (0.270 g, 1 mmol) and di(acetylacetonato)dioxomolybdenum(VI) (0.328 g, 1 mmol) were dissolved in heated in methanol for an hour. To the orange solution was added 4,4'-bipyridine (0.08 g, 0.5 mmol); heating was continued for another hour. The solution was filtered and set aside for the growth of crystals, m.p. 495–497 K.

### Refinement

Carbon-bound H-atoms were placed in calculated positions (C—H 0.95 to 0.99 Å) and were included in the refinement in the riding model approximation, with *U*(H) set to 1.2 times *U*_eq_(C).

The water H-atoms were located in a difference Fourier map and were refined with distance restraints of O–H 0.84±0.01 and H···H 1.37±0.01 Å; their temperature factors were refined.

The 4,4'-bipyridine molecule is disordered about a center-of-inversion. The pyridyl ring was refined as two rings that shared common N and and C*_para_* atoms. As the occupancy refined to nearly 1/2, the occupancy was then fixed as 0.5. Carbon–nitrogen distances were restrained to 1.35±0.01 Å and carbon–carbon distances to 1.39±0.01 Å. The six atoms of each ring were restrained to lie on a plane. Attempts to refined the disordered atoms anisotropically led to non-positive definites; the eight disordered atoms were then refined only isotropically.

Omitted from the refinement owing to bad disagreement were these reflections: (0 0 1), (-6 - 6 2), (4 - 5 4) and (3 9 7).

### Crystal data

**Table:**

[Mo(C_14_H_12_N_2_O_4_)O_2_(H_2_O)]·0.5C_10_H_8_N_2_	*Z* = 2
*M**_r_* = 496.30	*F*(000) = 502
Triclinic, *P*1	*D*_x_ = 1.685 Mg m^−^^3^
Hall symbol: -P 1	Mo *K*α radiation, λ = 0.71073 Å
*a* = 7.9237 (1) Å	Cell parameters from 8273 reflections
*b* = 10.1869 (1) Å	θ = 2.5–28.2°
*c* = 13.3215 (2) Å	µ = 0.72 mm^−^^1^
α = 78.7841 (5)°	*T* = 100 K
β = 78.4605 (5)°	Block, orange
γ = 69.5728 (5)°	0.2 × 0.2 × 0.2 mm
*V* = 978.15 (2) Å^3^	

### Data collection

**Table:**

Bruker SMART APEX diffractometer	4445 independent reflections
Radiation source: fine-focus sealed tube	4266 reflections with *I* > 2σ(*I*)
graphite	*R*_int_ = 0.019
ω scans	θ_max_ = 27.5°, θ_min_ = 2.2°
Absorption correction: multi-scan (*SADABS*; Sheldrick, 1996)	*h* = −10→10
*T*_min_ = 0.649, *T*_max_ = 0.746	*k* = −13→13
9175 measured reflections	*l* = −17→17

### Refinement

**Table:**

Refinement on *F*^2^	Primary atom site location: structure-invariant direct methods
Least-squares matrix: full	Secondary atom site location: difference Fourier map
*R*[*F*^2^ > 2σ(*F*^2^)] = 0.026	Hydrogen site location: inferred from neighbouring sites
*wR*(*F*^2^) = 0.076	H atoms treated by a mixture of independent and constrained refinement
*S* = 0.98	*w* = 1/[σ^2^(*F*_o_^2^) + (0.0474*P*)^2^ + 1.0897*P*] where *P* = (*F*_o_^2^ + 2*F*_c_^2^)/3
4445 reflections	(Δ/σ)_max_ = 0.001
275 parameters	Δρ_max_ = 0.73 e Å^−^^3^
24 restraints	Δρ_min_ = −0.72 e Å^−^^3^

### Fractional atomic coordinates and isotropic or equivalent isotropic displacement parameters (Å^2^)

**Table:**

	*x*	*y*	*z*	*U*_iso_*/*U*_eq_	Occ. (<1)
Mo1	0.74598 (2)	0.304217 (17)	0.170071 (12)	0.01695 (7)	
O1	1.1355 (2)	0.30269 (16)	−0.20486 (13)	0.0246 (3)	
O2	0.9252 (2)	0.21432 (15)	0.05147 (12)	0.0195 (3)	
O3	0.6036 (2)	0.46573 (17)	0.23935 (12)	0.0218 (3)	
O4	0.3337 (2)	0.5786 (2)	0.37565 (13)	0.0304 (4)	
O5	0.8232 (2)	0.17630 (17)	0.26946 (12)	0.0224 (3)	
O6	0.5697 (2)	0.26804 (17)	0.13977 (12)	0.0223 (3)	
O1W	0.9855 (2)	0.36664 (17)	0.18299 (12)	0.0210 (3)	
H11	1.047 (3)	0.327 (3)	0.2310 (16)	0.028 (8)*	
H12	1.017 (4)	0.435 (2)	0.150 (2)	0.043 (9)*	
N1	0.8802 (2)	0.43227 (18)	−0.05053 (13)	0.0173 (3)	
N2	0.7547 (2)	0.47579 (18)	0.03625 (13)	0.0163 (3)	
N3	1.1836 (3)	0.2227 (2)	0.33511 (17)	0.0305 (5)	
C1	1.2652 (3)	0.2079 (3)	−0.26360 (19)	0.0270 (5)	
H1	1.3224	0.2325	−0.3310	0.032*	
C2	1.3007 (3)	0.0757 (3)	−0.2131 (2)	0.0275 (5)	
H2	1.3847	−0.0080	−0.2377	0.033*	
C3	1.1882 (3)	0.0854 (2)	−0.11569 (18)	0.0229 (4)	
H3	1.1818	0.0099	−0.0622	0.027*	
C4	1.0916 (3)	0.2245 (2)	−0.11444 (16)	0.0193 (4)	
C5	0.9575 (3)	0.2954 (2)	−0.03452 (16)	0.0176 (4)	
C6	0.6621 (3)	0.6087 (2)	0.02969 (16)	0.0179 (4)	
H6	0.6873	0.6688	−0.0314	0.022*	
C7	0.5219 (3)	0.6710 (2)	0.11060 (16)	0.0197 (4)	
C8	0.4088 (3)	0.8115 (2)	0.08794 (18)	0.0240 (4)	
H8	0.4312	0.8640	0.0224	0.029*	
C9	0.2662 (3)	0.8737 (3)	0.1600 (2)	0.0297 (5)	
H9	0.1900	0.9680	0.1436	0.036*	
C10	0.2339 (3)	0.7978 (3)	0.25712 (19)	0.0304 (5)	
H10	0.1334	0.8398	0.3059	0.036*	
C11	0.3482 (3)	0.6612 (3)	0.28256 (18)	0.0252 (5)	
C12	0.4943 (3)	0.5964 (2)	0.20924 (16)	0.0207 (4)	
C13	0.1903 (3)	0.6364 (3)	0.45495 (19)	0.0340 (6)	
H13A	0.0704	0.6616	0.4318	0.041*	
H13B	0.2028	0.7225	0.4722	0.041*	
C14	0.2070 (4)	0.5235 (4)	0.5478 (2)	0.0386 (6)	
H14A	0.1101	0.5580	0.6041	0.058*	
H14B	0.3257	0.5005	0.5703	0.058*	
H14C	0.1960	0.4385	0.5293	0.058*	
C15	1.2028 (5)	0.0903 (4)	0.3618 (3)	0.0214 (8)*	0.50
H15	1.1241	0.0543	0.3384	0.026*	0.50
C16	1.3288 (5)	−0.0028 (4)	0.4216 (3)	0.0206 (8)*	0.50
H16	1.3433	−0.1009	0.4325	0.025*	0.50
C15'	1.1327 (6)	0.1138 (4)	0.4102 (3)	0.0199 (8)*	0.50
H15'	1.0185	0.1015	0.4124	0.024*	0.50
C16'	1.2513 (5)	0.0266 (4)	0.4794 (3)	0.0208 (8)*	0.50
H16'	1.2182	−0.0413	0.5320	0.025*	0.50
C17	1.4335 (4)	0.0471 (2)	0.4655 (2)	0.0340 (6)	
C18	1.4347 (8)	0.1850 (6)	0.4256 (5)	0.0223 (19)*	0.50
H18	1.5229	0.2190	0.4409	0.027*	0.50
C19	1.3061 (8)	0.2709 (7)	0.3637 (4)	0.0284 (19)*	0.50
H19	1.3025	0.3660	0.3403	0.034*	0.50
C18'	1.4505 (8)	0.1689 (6)	0.4113 (5)	0.0148 (14)*	0.50
H18'	1.5499	0.1977	0.4166	0.018*	0.50
C19'	1.3228 (6)	0.2528 (5)	0.3477 (4)	0.0116 (11)*	0.50
H19'	1.3389	0.3383	0.3109	0.014*	0.50

### Atomic displacement parameters (Å^2^)

**Table:**

	*U*^11^	*U*^22^	*U*^33^	*U*^12^	*U*^13^	*U*^23^
Mo1	0.01730 (11)	0.01891 (11)	0.01575 (11)	−0.00872 (7)	−0.00505 (7)	0.00321 (7)
O1	0.0285 (8)	0.0188 (7)	0.0251 (8)	−0.0096 (6)	0.0024 (6)	−0.0023 (6)
O2	0.0230 (7)	0.0158 (7)	0.0197 (7)	−0.0081 (6)	−0.0036 (6)	0.0017 (5)
O3	0.0227 (7)	0.0251 (8)	0.0170 (7)	−0.0075 (6)	−0.0037 (6)	−0.0008 (6)
O4	0.0255 (8)	0.0428 (10)	0.0201 (8)	−0.0114 (8)	0.0031 (6)	−0.0039 (7)
O5	0.0226 (7)	0.0246 (8)	0.0220 (7)	−0.0124 (6)	−0.0085 (6)	0.0068 (6)
O6	0.0212 (7)	0.0279 (8)	0.0204 (7)	−0.0117 (6)	−0.0057 (6)	0.0007 (6)
O1W	0.0224 (8)	0.0227 (8)	0.0212 (7)	−0.0133 (6)	−0.0097 (6)	0.0074 (6)
N1	0.0178 (8)	0.0179 (8)	0.0163 (8)	−0.0066 (7)	−0.0023 (6)	−0.0013 (6)
N2	0.0162 (8)	0.0193 (8)	0.0140 (8)	−0.0062 (7)	−0.0041 (6)	−0.0011 (6)
N3	0.0383 (12)	0.0186 (9)	0.0381 (12)	−0.0039 (8)	−0.0241 (9)	−0.0027 (8)
C1	0.0269 (11)	0.0288 (11)	0.0255 (11)	−0.0111 (9)	0.0035 (9)	−0.0081 (9)
C2	0.0260 (11)	0.0220 (11)	0.0355 (13)	−0.0066 (9)	−0.0012 (9)	−0.0116 (9)
C3	0.0216 (10)	0.0179 (10)	0.0285 (11)	−0.0063 (8)	−0.0049 (8)	−0.0006 (8)
C4	0.0196 (9)	0.0186 (10)	0.0217 (10)	−0.0087 (8)	−0.0045 (8)	−0.0010 (8)
C5	0.0177 (9)	0.0182 (9)	0.0192 (9)	−0.0082 (8)	−0.0062 (7)	0.0002 (7)
C6	0.0191 (9)	0.0193 (9)	0.0165 (9)	−0.0071 (8)	−0.0065 (7)	0.0006 (7)
C7	0.0183 (9)	0.0222 (10)	0.0198 (10)	−0.0054 (8)	−0.0063 (8)	−0.0046 (8)
C8	0.0235 (11)	0.0244 (11)	0.0223 (10)	−0.0030 (9)	−0.0078 (8)	−0.0036 (8)
C9	0.0230 (11)	0.0308 (12)	0.0308 (12)	0.0022 (9)	−0.0088 (9)	−0.0091 (10)
C10	0.0195 (10)	0.0425 (14)	0.0272 (12)	−0.0035 (10)	−0.0025 (9)	−0.0133 (10)
C11	0.0204 (10)	0.0371 (13)	0.0209 (10)	−0.0115 (9)	−0.0031 (8)	−0.0061 (9)
C12	0.0185 (10)	0.0252 (10)	0.0203 (10)	−0.0077 (8)	−0.0053 (8)	−0.0039 (8)
C13	0.0256 (12)	0.0534 (16)	0.0240 (11)	−0.0151 (11)	0.0054 (9)	−0.0122 (11)
C14	0.0331 (14)	0.0623 (19)	0.0219 (12)	−0.0197 (13)	0.0026 (10)	−0.0080 (12)
C17	0.0435 (15)	0.0168 (10)	0.0499 (16)	−0.0094 (10)	−0.0341 (13)	0.0040 (10)

### Geometric parameters (Å, °)

**Table:**

Mo1—O6	1.7007 (15)	C7—C8	1.410 (3)
Mo1—O5	1.7093 (15)	C8—C9	1.379 (3)
Mo1—O3	1.9262 (16)	C8—H8	0.9500
Mo1—O2	2.0270 (15)	C9—C10	1.398 (4)
Mo1—O1W	2.2479 (15)	C9—H9	0.9500
Mo1—N2	2.2495 (17)	C10—C11	1.389 (4)
O1—C4	1.364 (3)	C10—H10	0.9500
O1—C1	1.371 (3)	C11—C12	1.413 (3)
O2—C5	1.313 (2)	C13—C14	1.509 (4)
O3—C12	1.345 (3)	C13—H13A	0.9900
O4—C11	1.365 (3)	C13—H13B	0.9900
O4—C13	1.431 (3)	C14—H14A	0.9800
O1W—H11	0.833 (10)	C14—H14B	0.9800
O1W—H12	0.837 (11)	C14—H14C	0.9800
N1—C5	1.305 (3)	C15—C16	1.377 (5)
N1—N2	1.398 (2)	C15—H15	0.9500
N2—C6	1.291 (3)	C16—C17	1.376 (4)
N3—C15	1.288 (4)	C16—H16	0.9500
N3—C19'	1.291 (5)	C15'—C16'	1.403 (5)
N3—C19	1.371 (6)	C15'—H15'	0.9500
N3—C15'	1.454 (4)	C16'—C17	1.500 (5)
C1—C2	1.344 (4)	C16'—H16'	0.9500
C1—H1	0.9500	C17—C18'	1.345 (5)
C2—C3	1.419 (3)	C17—C18	1.405 (6)
C2—H2	0.9500	C17—C17^i^	1.487 (4)
C3—C4	1.356 (3)	C18—C19	1.386 (7)
C3—H3	0.9500	C18—H18	0.9500
C4—C5	1.448 (3)	C19—H19	0.9500
C6—C7	1.447 (3)	C18'—C19'	1.389 (6)
C6—H6	0.9500	C18'—H18'	0.9500
C7—C12	1.402 (3)	C19'—H19'	0.9500
			
O6—Mo1—O5	105.24 (7)	C8—C9—H9	120.0
O6—Mo1—O3	97.40 (7)	C10—C9—H9	120.0
O5—Mo1—O3	103.06 (7)	C11—C10—C9	120.2 (2)
O6—Mo1—O2	93.86 (7)	C11—C10—H10	119.9
O5—Mo1—O2	98.70 (7)	C9—C10—H10	119.9
O3—Mo1—O2	151.84 (6)	O4—C11—C10	125.6 (2)
O6—Mo1—O1W	170.71 (7)	O4—C11—C12	114.2 (2)
O5—Mo1—O1W	82.87 (6)	C10—C11—C12	120.2 (2)
O3—Mo1—O1W	84.91 (6)	O3—C12—C7	123.18 (19)
O2—Mo1—O1W	80.25 (6)	O3—C12—C11	117.5 (2)
O6—Mo1—N2	95.98 (7)	C7—C12—C11	119.3 (2)
O5—Mo1—N2	157.48 (7)	O4—C13—C14	106.5 (2)
O3—Mo1—N2	81.20 (6)	O4—C13—H13A	110.4
O2—Mo1—N2	71.99 (6)	C14—C13—H13A	110.4
O1W—Mo1—N2	75.43 (6)	O4—C13—H13B	110.4
C4—O1—C1	105.58 (18)	C14—C13—H13B	110.4
C5—O2—Mo1	118.96 (13)	H13A—C13—H13B	108.6
C12—O3—Mo1	134.52 (14)	C13—C14—H14A	109.5
C11—O4—C13	118.0 (2)	C13—C14—H14B	109.5
Mo1—O1W—H11	120.4 (18)	H14A—C14—H14B	109.5
Mo1—O1W—H12	128.8 (19)	C13—C14—H14C	109.5
H11—O1W—H12	110.1 (17)	H14A—C14—H14C	109.5
C5—N1—N2	109.13 (17)	H14B—C14—H14C	109.5
C6—N2—N1	115.97 (17)	N3—C15—C16	124.9 (4)
C6—N2—Mo1	128.78 (14)	N3—C15—H15	117.5
N1—N2—Mo1	115.24 (12)	C16—C15—H15	117.5
C15—N3—C19	116.8 (4)	C17—C16—C15	119.5 (4)
C19'—N3—C15'	117.2 (3)	C17—C16—H16	120.2
C2—C1—O1	110.9 (2)	C15—C16—H16	120.2
C2—C1—H1	124.5	C16'—C15'—N3	120.4 (4)
O1—C1—H1	124.5	C16'—C15'—H15'	119.8
C1—C2—C3	106.6 (2)	N3—C15'—H15'	119.8
C1—C2—H2	126.7	C15'—C16'—C17	116.0 (3)
C3—C2—H2	126.7	C15'—C16'—H16'	122.0
C4—C3—C2	106.1 (2)	C17—C16'—H16'	122.0
C4—C3—H3	126.9	C16—C17—C18	115.8 (3)
C2—C3—H3	126.9	C18'—C17—C17^i^	122.4 (3)
C3—C4—O1	110.83 (19)	C16—C17—C17^i^	122.2 (3)
C3—C4—C5	130.1 (2)	C18—C17—C17^i^	120.9 (3)
O1—C4—C5	119.02 (18)	C18'—C17—C16'	117.6 (3)
N1—C5—O2	124.54 (19)	C17^i^—C17—C16'	117.9 (3)
N1—C5—C4	119.62 (19)	C19—C18—C17	119.3 (5)
O2—C5—C4	115.83 (18)	C19—C18—H18	120.4
N2—C6—C7	123.35 (19)	C17—C18—H18	120.4
N2—C6—H6	118.3	N3—C19—C18	121.9 (6)
C7—C6—H6	118.3	N3—C19—H19	119.1
C12—C7—C8	119.5 (2)	C18—C19—H19	119.1
C12—C7—C6	122.4 (2)	C17—C18'—C19'	120.1 (4)
C8—C7—C6	118.11 (19)	C17—C18'—H18'	119.9
C9—C8—C7	120.7 (2)	C19'—C18'—H18'	119.9
C9—C8—H8	119.7	N3—C19'—C18'	124.6 (4)
C7—C8—H8	119.7	N3—C19'—H19'	117.7
C8—C9—C10	120.0 (2)	C18'—C19'—H19'	117.7
			
O6—Mo1—O2—C5	97.28 (15)	C13—O4—C11—C12	179.4 (2)
O5—Mo1—O2—C5	−156.65 (15)	C9—C10—C11—O4	177.9 (2)
O3—Mo1—O2—C5	−16.3 (2)	C9—C10—C11—C12	−2.1 (4)
O1W—Mo1—O2—C5	−75.49 (14)	Mo1—O3—C12—C7	−32.0 (3)
N2—Mo1—O2—C5	2.25 (14)	Mo1—O3—C12—C11	149.69 (17)
O6—Mo1—O3—C12	−66.0 (2)	C8—C7—C12—O3	−175.3 (2)
O5—Mo1—O3—C12	−173.64 (19)	C6—C7—C12—O3	5.3 (3)
O2—Mo1—O3—C12	46.7 (3)	C8—C7—C12—C11	3.0 (3)
O1W—Mo1—O3—C12	104.91 (19)	C6—C7—C12—C11	−176.4 (2)
N2—Mo1—O3—C12	28.90 (19)	O4—C11—C12—O3	−2.0 (3)
C5—N1—N2—C6	−177.34 (18)	C10—C11—C12—O3	178.0 (2)
C5—N1—N2—Mo1	3.9 (2)	O4—C11—C12—C7	179.61 (19)
O6—Mo1—N2—C6	85.90 (18)	C10—C11—C12—C7	−0.3 (3)
O5—Mo1—N2—C6	−113.6 (2)	C11—O4—C13—C14	180.0 (2)
O3—Mo1—N2—C6	−10.70 (18)	C19'—N3—C15—C16	16.4 (3)
O2—Mo1—N2—C6	178.02 (19)	C19—N3—C15—C16	4.2 (3)
O1W—Mo1—N2—C6	−97.69 (18)	C15'—N3—C15—C16	−89.7 (5)
O6—Mo1—N2—N1	−95.50 (14)	N3—C15—C16—C17	6.3 (3)
O5—Mo1—N2—N1	65.0 (2)	C15—N3—C15'—C16'	76.3 (5)
O3—Mo1—N2—N1	167.90 (14)	C19'—N3—C15'—C16'	−12.7 (5)
O2—Mo1—N2—N1	−3.38 (12)	C19—N3—C15'—C16'	−25.7 (5)
O1W—Mo1—N2—N1	80.91 (13)	N3—C15'—C16'—C17	−4.0 (5)
C4—O1—C1—C2	0.5 (3)	C15—C16—C17—C18'	−25.2 (4)
O1—C1—C2—C3	−0.4 (3)	C15—C16—C17—C18	−14.9 (4)
C1—C2—C3—C4	0.1 (3)	C15—C16—C17—C17^i^	177.4 (3)
C2—C3—C4—O1	0.2 (3)	C15—C16—C17—C16'	82.9 (4)
C2—C3—C4—C5	−179.5 (2)	C15'—C16'—C17—C18'	18.8 (6)
C1—O1—C4—C3	−0.4 (2)	C15'—C16'—C17—C16	−70.2 (4)
C1—O1—C4—C5	179.28 (19)	C15'—C16'—C17—C18	30.1 (5)
N2—N1—C5—O2	−2.2 (3)	C15'—C16'—C17—C17^i^	−177.6 (3)
N2—N1—C5—C4	179.02 (17)	C18'—C17—C18—C19	80 (2)
Mo1—O2—C5—N1	−0.9 (3)	C16—C17—C18—C19	13.7 (6)
Mo1—O2—C5—C4	177.96 (13)	C17^i^—C17—C18—C19	−178.5 (5)
C3—C4—C5—N1	−179.8 (2)	C16'—C17—C18—C19	−27.0 (6)
O1—C4—C5—N1	0.6 (3)	C15—N3—C19—C18	−5.3 (6)
C3—C4—C5—O2	1.3 (3)	C19'—N3—C19—C18	−76 (2)
O1—C4—C5—O2	−178.33 (18)	C15'—N3—C19—C18	30.5 (6)
N1—N2—C6—C7	177.65 (18)	C17—C18—C19—N3	−3.8 (7)
Mo1—N2—C6—C7	−3.8 (3)	C16—C17—C18'—C19'	22.4 (7)
N2—C6—C7—C12	10.2 (3)	C18—C17—C18'—C19'	−95 (2)
N2—C6—C7—C8	−169.2 (2)	C17^i^—C17—C18'—C19'	179.8 (5)
C12—C7—C8—C9	−3.2 (3)	C16'—C17—C18'—C19'	−17.4 (7)
C6—C7—C8—C9	176.2 (2)	C15—N3—C19'—C18'	−19.5 (7)
C7—C8—C9—C10	0.8 (4)	C19—N3—C19'—C18'	95 (2)
C8—C9—C10—C11	1.9 (4)	C15'—N3—C19'—C18'	15.7 (7)
C13—O4—C11—C10	−0.6 (3)	C17—C18'—C19'—N3	−0.3 (9)

Symmetry codes: (i) −*x*+3, −*y*, −*z*+1.

### Hydrogen-bond geometry (Å, °)

**Table:**

*D*—H···*A*	*D*—H	H···*A*	*D*···*A*	*D*—H···*A*
O1w—H11···N3	0.83 (1)	1.86 (1)	2.689 (3)	174 (3)
O1w—H12···N1^ii^	0.84 (1)	1.97 (1)	2.794 (2)	167 (3)

Symmetry codes: (ii) −*x*+2, −*y*+1, −*z*.
